# Potential of New Sustainable Green Geopolymer Metal Composite (GGMC) Material as Mould Insert for Rapid Tooling (RT) in Injection Moulding Process

**DOI:** 10.3390/ma16041724

**Published:** 2023-02-19

**Authors:** Allice Tan Mun Yin, Shayfull Zamree Abd Rahim, Mohd Mustafa Al Bakri Abdullah, Marcin Nabialek, Abdellah El-hadj Abdellah, Allan Rennie, Muhammad Faheem Mohd Tahir, Aurel Mihail Titu

**Affiliations:** 1 Faculty of Mechanical Engineering & Technology, Universiti Malaysia Perlis, Arau 02600, Malaysia; 2 Center of Excellence Geopolymer and Green Technology (CEGeoGTech), Universiti Malaysia Perlis, Kangar 01000, Malaysia; 3 Faculty of Chemical Engineering & Technology, Universiti Malaysia Perlis, Kangar 01000, Malaysia; 4 Department of Physics, Faculty of Production Engineering and Materials Technology, Częstochowa University of Technology, 42-201 Czestochowa, Poland; 5 Laboratory of Mechanics, Physics and Mathematical Modelling (LMP2M), University of Medea, Medea 26000, Algeria; 6 Lancaster Product Development Unit, Engineering Department, Lancaster University, Lancaster LA1 4YW, UK; 7 Industrial Engineering and Management Department, Faculty of Engineering, “Lucian Blaga” University of Sibiu, 10 Victoriei Street, 550024 Sibiu, Romania

**Keywords:** rapid tooling, geopolymer metal composite, additive manufacturing, injection moulding process

## Abstract

The investigation of mould inserts in the injection moulding process using metal epoxy composite (MEC) with pure metal filler particles is gaining popularity among researchers. Therefore, to attain zero emissions, the idea of recycling metal waste from industries and workshops must be investigated (waste free) because metal recycling conserves natural resources while requiring less energy to manufacture new products than virgin raw materials would. The utilisation of metal scrap for rapid tooling (RT) in the injection moulding industry is a fascinating and potentially viable approach. On the other hand, epoxy that can endure high temperatures (>220 °C) is challenging to find and expensive. Meanwhile, industrial scrap from coal-fired power plants can be a precursor to creating geopolymer materials with desired physical and mechanical qualities for RT applications. One intriguing attribute of geopolymer is its ability to endure temperatures up to 1000 °C. Nonetheless, geopolymer has a higher compressive strength of 60–80 MPa (8700–11,600 psi) than epoxy (68.95 MPa) (10,000 psi). Aside from its low cost, geopolymer offers superior resilience to harsh environments and high compressive and flexural strength. This research aims to investigate the possibility of generating a new sustainable material by integrating several types of metals in green geopolymer metal composite (GGMC) mould inserts for RT in the injection moulding process. It is necessary to examine and investigate the optimal formulation of GGMC as mould inserts for RT in the injection moulding process. With less expensive and more ecologically friendly components, the GGMC is expected to be a superior choice as a mould insert for RT. This research substantially impacts environmental preservation, cost reduction, and maintaining and sustaining the metal waste management system. As a result of the lower cost of recycled metals, sectors such as mould-making and machining will profit the most.

## 1. Introduction

Time to market is a crucial aspect of a product development strategy, and speed is frequently compared to other factors such as functionality, creativity, or performance [1,2,3]. With numerous new technologies, worldwide rivalry for product creation is soaring. Furthermore, companies are always looking for cutting-edge technologies that are cost-effective, capable of manufacturing goods in tiny quantities while maintaining excellent performance, and able to meet sustainability goals. This has driven the development of rapid tooling (RT) techniques, which are needed in today’s market to replace traditional techniques with rapid product innovation and improve manufacturing processes, particularly mould-making [4,5,6].

**Figure 1:**
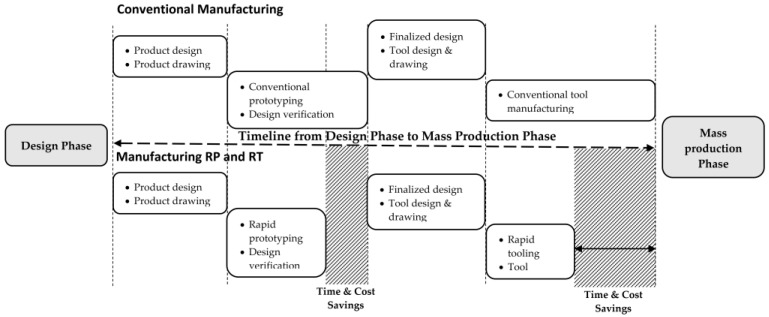
A Review of the Application of Rapid Tooling in Manufacturing [8].

As shown in Figure 1, RT provides quicker manufacturing for completing tests and starting final production, minimises costs, and reduces project time [7].

Every industry, regardless of size, experiences a time when rapid tooling is required to address particular problems. Additionally, an improved tooling system is required for creating a limited number of functional prototypes to assess the product development cycle [8,9,10]. A small quantity of items is often utilised as a marketplace trial, evaluation need, and manufacturing process design [9,10].

Before mass manufacturing, functioning tools or prototypes must be launched for every scientific study [11,12,13,14]. These are not made available in large quantities to consumers but rather in limited amounts to researchers. RT is highly advantageous in this circumstance since it allows for the rapid introduction of items. Furthermore, the uses of production tools allow mass production to be obtained at a lower price because manufacturing costs are cheap. For this reason, many brand-new businesses and even big organisations prefer this technology to boost their profits and obtain a market advantage over their rivals [1,2,3].

Prototype companies or mould producers typically employ mild steel or aluminium for the mould inserts in RT. Production toolmaking is time-consuming and costly, and machining involves the same computer numerical control (CNC), electrical discharge machining (EDM), and electric discharge machining (wire EDM) procedures [15,16]. Recently, additive manufacturing (AM) has been employed to create mould inserts for RT [13,16]. For a limited number of prototypes, RT often uses models or prototypes made by AM as templates for manufacturing mould inserts or uses the AM process directly [4,5,6]. Numerous RT technologies are available on the market, such as a hybrid technique combining RT and AM to shorten RT production time.

RT can be categorised as either an indirect or direct technique and differs from traditional tooling in that the amount of time needed to create the tooling is significantly reduced [17,18]. Automated manufacturing methods use the AM process to generate mould inserts without the requirement for values to be predicted. Direct tooling includes processes such as additive manufacturing (AM), stereolithography (SLA), jet photopolymerisation (PolyJet), fused deposition moulding (FDM), and selective laser sintering (SLS) [5,6,19]. Alternatively, the AM project as a master model is considered a secondary approach to create moulds for casting or plastic moulding processes. This technique combines the 3D KelTool process, metal casting, plastic casting, elastic moulding, and other comparable procedures to create injection moulding inserts [5,6,15,20]. Inserts constructed from epoxy-acrylate and utilising material for injection moulding by homo-polypropylene are used for quick tooling applications for 3D-printed injection moulds [21]. The mould insert constructed from steel and copper for hybrid prototype mould applications is created using a mix of laser powder bed fusion (L-PBF) and casting. In a study, L-PBF printed the steel shell with conformal cooling channels, and the shell was cast with copper [22]. For a material jetting (PolyJet) mould, a mould insert constructed from epoxy-acrylate resin is used and its in-mould behaviour is compared to a guidance mould insert fabricated from aluminium [23].

Failure in the moulding process in generating RT mould inserts is common for technologies with poor thermal and mechanical quality, such as SLS, FDM, SLA, PolyJet, and AM [5,6,19]. Zakzewski et al. [24] presented the bucking π-theorem, which was modified to analyse and characterise the poor surface quality Ra issue of SLS/SLM-processed samples, as well as the existence of porosity, a material structure defect. Furthermore, the use of SLA models is physically restricted. These constraints can be solved by developing procedures that use SLA parts as the “master blueprint” for the silicone mould process. In comparison to mechanical techniques, the DMLS process is inefficient for the design of basic plastic components. Furthermore, DMLS imposes a few constraints for a specific feature design for complicated components. According to previous studies on RT mould inserts, the stress applied to the mould insert during the injection cycle has a significant influence on the mould life [11,15,19,20]. Nowadays, a combination of RP technique and production tooling helps produce RT more quickly but faces dimensional accuracy and surface finish issues [17,18]. Moreover, the injection moulding process faces a cooling time issue where most of the mould inserts fabricated using RP techniques have very low thermal conductivity; thus, increasing the cooling rate will undoubtedly influence the cycle time for producing the components [4,16,25,26]. One of the RT options to increase competitiveness is using metal epoxy composite (MEC), which provides greater heat conductivity as mould inserts in RT application and lowers tooling production costs and lead times by 25% and 50%, respectively [3,6]. Using optimisation methods to determine the optimal composition for materials, as recommended in the linked literature, can be considered for future research, such as determining the best amount of Al or Cu to mix with epoxy resin for desirable mechanical properties [27,28,29,30,31,32,33,34,35,36,37]. The use of MEC mould inserts for RT in the injection moulding process, which uses pure metal filler particles combined with epoxy resin, has attracted the attention of many researchers [20,29,38,39,40].

The use of MEC materials as mould inserts offers better thermal and mechanical properties as compared to mould inserts produced using AM technologies [40,41,42,43]. However, dimensional accuracy and surface quality still need to be improved, so after the fabrication of mould inserts using MEC material and casting techniques, the mould inserts need to go through a secondary process (machining) to improve mould dimensional precision and surface quality in terms of cavities, especially for precision of plastic products (±0.05 mm).

Secondary (recycled) materials compete with primary materials in the metals business. Primary materials require the use of finite resources. Producing scrap materials or processed secondary metals can sometimes be more cost-effective than producing new primary materials, provided that the cost of collecting the waste is not prohibitively expensive [42]. Due to its spherical morphology and manageable particle size dispersion, gas-atomised (GA) powder is the most frequent feedstock for AM. However, much energy and inert gas are required to make GA powders [43]. Water-atomised powder is another option when increased powder solidification rates and reduced manufacturing costs are the priorities [29,44,45]. In contrast, melting the metal before ejection from the atomisation nozzles is energy-intensive because of the significant enthalpy difference between the liquid and solid states [45]. In metal AM, the feedstock powder is often remelted. This repeated melting is a costly and inefficient process [46]. As it can reduce materials of varying sizes to powder, mechanical milling offers a chance for environmentally friendly powder production [31,47,48,49]. For mechanical milling, ambient or cryogenic temperatures are typically used [49,50]. The aforementioned considerable energy input is no longer required to reach atomisation temperatures [50]. Due to these potential benefits, mechanical milling is being used to reduce metal machining chips to powders that can be used in AM [46].

On the other hand, Davidovits’ geopolymer technology is one of the groundbreaking innovations resulting in an affordable and greener binder alternative. The silica and aluminium in geosource materials such as metakaolin (calcined kaolin), and maybe techniques such as fly ash and bottom ash, are combined with the alkaline liquid to generate a geopolymer, an alkali-activated binder [1,11]. As a result, it reduces not only CO_2_ emissions but also recycles industrial waste, specifically using an aluminium–silicate mix to create products of higher value [9,10]. MEC using pure metal filler particles is beginning to be used by some researchers to investigate mould inserts in the injection moulding process [4,5,7]. However, a type of epoxy that can withstand high temperatures (>220 °C) is hard to find and still costly.

Additionally, besides municipal solid waste, coal combustion production (CCP) has been identified as the second-largest pollutant in the world. In 2011, about 130 metric tonnes (MT) of CCP were generated, with only 56.57 MT (43.50%) effectively used [51].

The four forms of solid waste created in substantial amounts by the CCP are boiler slag, bottom ash, fly ash, and flue gas desulphurisation (FGD) material [40,41,42]. One hundred and thirty metric tonnes of CCP included around 59.9 MT of fly ash. Fly ash was disposed of in surface impoundments covered with compacted clay soil, a plastic sheet, or both for the remaining 22.9 MT (38.36%) in landfills or surface impoundments [51,52,53]. The Environmental Protection Agency (EPA) of the United States (US) is now investigating the positive uses of fly ash [53,54,55,56,57]. This eliminates major health concerns associated with heavy metals and radioactive elements accumulated from fly ash disposal over time. Geopolymers derived from environmentally friendly materials, such as slag, or industrial by-products and used as a binding material are known as “green material”.

One interesting property of geopolymer is that it can withstand temperatures up to 1000 °C. Nevertheless, geopolymer only has a compressive strength of 60–80 MPa (8700–11,600 psi), while epoxy has a compressive strength of 68.95 MPa (10,000 psi) [29,58]. However, employing geopolymer material has similar issues to using epoxy resin, which necessitates determining the optimal strength, accuracy, acceptable surface finish, and good thermal characteristics.

Early strength of geopolymer can be obtained as early as 1 day, with compressive strength up to 15 MPa, and continues increase up to 40–50MPa within 7 days, which is comparable with the strength offered by epoxy. Nevertheless, the optimum strength of geopolymer material can be obtained by 28 days (80 MPa) and the strength will keep on increasing over time [59].

It was recognised that the filler’s interlaminar strength controls the bond strength of geopolymer reinforced with filler. The fact that filler with a bigger particle size has a lower binding strength is also well known. In addition, compared to epoxy resin, geopolymer showed high bond strength for both wet and dry interface surface conditions [59].

On the other hand, as the need for an environmentally friendly society grows, the quantity of waste material must be continually decreased. Hence, in order to achieve zero emissions, the idea of recycling metal waste from factories and workshops needs to be examined (waste free) [60,61,62,63]. Metal recycling helps to conserve natural resources while requiring less energy for manufacturing new products than would be required for virgin raw materials. Waste-free recycling reduces the emission of carbon dioxide and certain other harmful gases while also saving money and enabling industrial companies to reduce their production costs [64,65].

Through a Google Patents search (https://patents.google.com/ accessed on 10 February 2023), six patents granted/published that make use of (1) metal composite and composites made of (2) geopolymer and (3) metal were located. Table 1 lists the search terms for this review’s related field, rapid tooling. Fibre-reinforced metal composites (aluminium matrix composites) were developed by Yamamoto et al. [65] using aluminium alloy with 6–11 wt. % nickel as the metal matrix and reinforcing fibres. To mass produce complex parts with near-net shapes, Behi et al. [66] proposed using steel tooling in an injection moulding machine. In comparison to more traditional methods, this approach to producing complicated metal tooling is relatively cost-effective, making it possible to rapidly fabricate complex shaped parts using normal metal, ceramic, and plastic processing. According to the metal matrix composite introduced by Shaikh et al. [67], the fibre to metal or alloy ratio ranges from about 9:1 to less than about 1:1, and the fibres have an average diameter of approximately eight micrometres with a coating. Amaya and Crounse [68] discovered rapid manufacturing of mould inserts by employing blank die inserts formed from material typically used in the metal injection moulding process of complex shaped components to achieve high machinability rates, time and cost savings, extended tool life, and material savings. The dry-mix composition, as proposed by Nematollahi and Sanjayan [69], includes (a) an aluminosilicate material rich in silica and alumina and (b) a powdered alkali activator. Moreover, the dry-mix composition is chosen so that (i) the SHGC may be generated at ambient temperature without liquid activator, and (ii) strain-hardening behaviour and multiple cracking behaviours are observed. A strain-hardened, ambient temperature-cured geopolymer composite (SHGC) is generated by adding water and using a method of manufacturing an ambient temperature-cured SHGC. Qiang et al. [70] proposed a geopolymer composite material that is a type of 3D print as well as their preparation technique and applications, which included blast furnace slag powder accounting for 20~25% of the total composition weight, steel-making slag powder accounting for 10~15%, fly ash accounting for 0~5%, mine tailing machine-made sand accounting for 33~45%, exciting composite agent accounting for 3~5%, high molecular weight polymer accounting for 2.5~3%, volume stabiliser accounting for 1~3%, thixotropic agent accounting for 1~2%, defoamer accounting for 0.05~0.1%, and mixing water accounting for 13.9~12.45%. Each component is stirred, and subsequently pumped into the 3D printer applications for construction. The present invention’s geopolymer composite material demonstrates good caking property, strong stability, good go-out pump from holding capacity and adhesive property, excellent form, and volume stability, resulting in the construction of buildings with good overall stability and safety during use. The six patents granted/published from 1990 to February 2023 are listed in Table 1.

**Figure 2:**
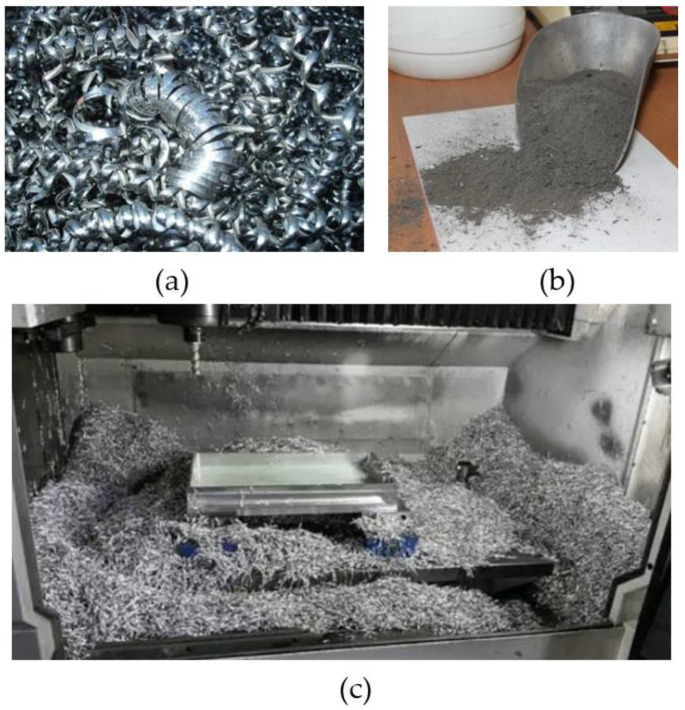
(**a**) Metal scraps from turning; (**b**) metal scraps from grinding; (**c**) metal scraps from milling.

RT is a cost-effective solution in the transition phase from new product development to mass production in the manufacturing industry [71,72]. RT, often referred to as bridge tooling, prototype tooling, or soft tooling, is a fast way to preproduce hundreds or even thousands of plastic parts prior to mass production, for design optimisation, functional testing, or preproduction verification, which can be a bridge between rapid prototyping (RP) and mass production. Shape, fit, and function prototype components are frequently made using RP technology, such as additive manufacturing [71,73,74]. Recycled metal waste such as mild steel, aluminium, copper, and brass after machining processes are as shown in Figure 2.

However, since 3D material qualities vary from those used in injection moulding, 3D-printed samples cannot provide a thorough evaluation of an injection-moulded part’s functional performance [18,59], making RT extremely crucial for the manufacturing industry.

**Figure 3:**
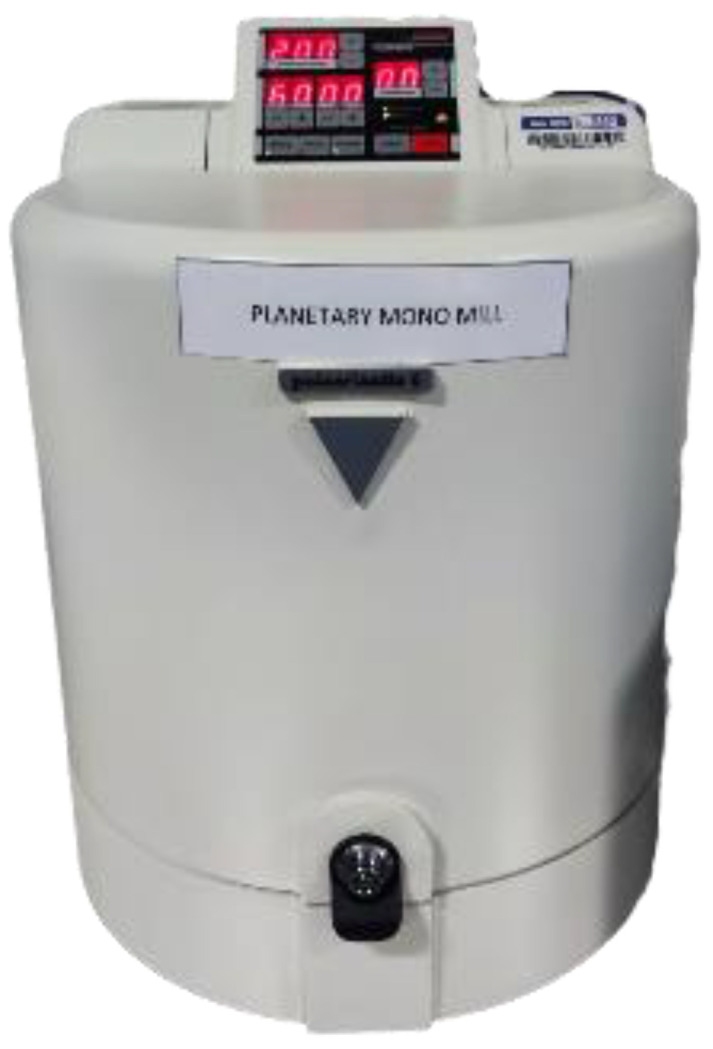
Planetary Mono Mill Pulverisette 6 can be used in the ball mill process.

**Figure 4:**
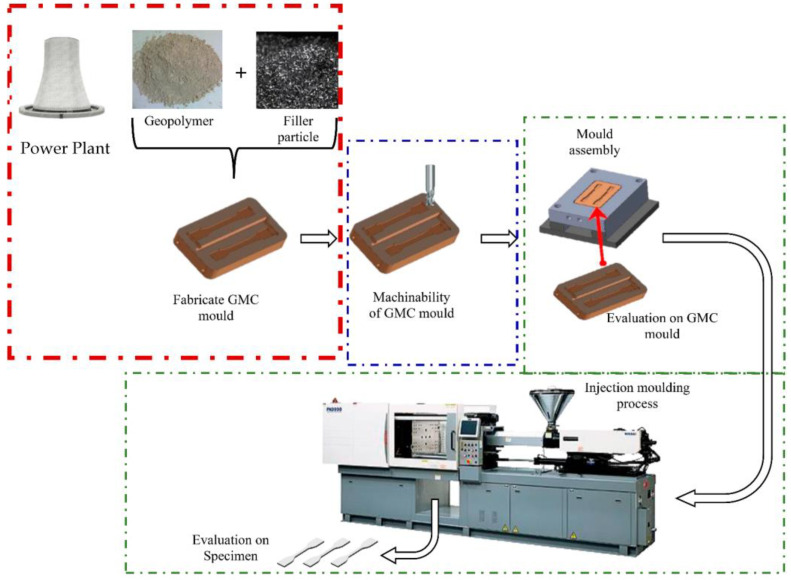
Graphical representation of GGMC as new material for mould inserts.

Using fly ash (waste from coal combustion) as the raw material, the metal scraps from the machining process are ground using a ball mill machine into a small and uniform size and mixed with geopolymer material to create green geopolymer metal composite (GGMC) material as in Figure 3. Then, this material can be used as mould inserts for RT applications which is expected to reduce tooling production costs and lead periods by up to 25% and 50%, respectively. The effect of GGMC material as mould inserts for RT in an injection moulding process and its relationship with compressive strength and thermal conductivity should be examined accordingly. Therefore, this research aims to determine whether geopolymer material may be used as RT mould inserts in the injection moulding process. The process by which GMCs are used as a new material for mould inserts is depicted in Figure 4.

A power plant is a structure that produces waste geopolymer and generates electric energy from another form of energy. The geopolymer material is then combined with filler particles (waste from machining after a ball milling process to form a powder filler). The ratio of geopolymer and powder filler is evaluated accordingly in terms of thermal conductivity and compressive strength. Next, the optimised ratio is used to fabricate the GMC mould inserts. Then, GMC mould inserts are machined accordingly to fit the insert size and assembled in the mould base. Following the examination of the GMC mould inserts, the GMC mould is assembled in the injection moulding machine to mould out the specimen for further evaluation of the mould parts’ quality in terms of shrinkage and warpage, including the cooling time required, which is definitely influenced by the thermal conductivity of the GMC mould inserts. The reliability of the GMC mould inserts is evaluated accordingly in terms of the number of shots (specimens) that can be produced before the mould starts to crack or wear.

## 2. Injection Moulding Process

### 2.1. Important Processing Parameters in the Injection Moulding Process

Processing parameters are essential to produce good-quality moulded parts in the injection moulding process. Previously, the trial-and-error approach to determine processing parameters relied upon a plastic injection moulding process. However, the trial-and-error approach is ineffective for complex manufacturing processes [75,76,77]. Therefore, many studies had been carried out over the years to minimise shrinkage and warpage defects by optimising the processing parameters [77,78,79,80]. In addition, it has also been observed that various critical processing factors, including packing pressure, melt temperature, packing shrinkage duration, mould temperature, and cooling time, have an impact on the quality of the moulded components produced (warpage) [77,81,82,83].

#### 2.1.1. Melt Temperature

Melt temperature is the temperature required to melt the plastic material in a pellet formed in the screw barrel of the injection moulding machine before the injection stage to fill the mould cavities [84,85]. Some researchers reported that melt temperature is a significant processing parameter that causes warpage defects on the moulded parts produced. The relationship between melt temperature and the flow of molten plastic into the mould cavities through feeding system has been studied and it was reported that the amount of material flow into the cavities is affected by the melt temperature [84,85].

#### 2.1.2. Cooling Time

When the molten plastic hits the walls of the mould cavities, it starts to cool down and continues to solidify. The mould stays closed until the moulded part reaches the ejection temperature. The part is ejected out from the injection mould once it becomes rigid enough [85,86]. When the cooling time, including that needed for the moulded component to achieve the injection temperature, is increased, shrinkage and warp issues are reduced [87,88]. However, the appropriate cooling time needs to be determined in order to produce moulded parts with good quality within the optimal cycle time.

#### 2.1.3. Packing Pressure

Packing pressure is the pressure used to inject and compress the molten plastic material into mould cavities until the gate freezes [85]. According to previous research, packing pressure is a crucial processing parameter that impacts the accuracy and quality of the moulded components produced. In addition, packing pressure is also a significant processing parameter after packing time which has a significant impact on shrinkage and flexural strength of the moulded parts produced [80,81,82]. Any changes in packing pressure will cause degradation of the mechanical properties of the parts moulded from virgin and recycled plastic material in various compositions. Inappropriate settings of packing pressure may result in high shrinkage defects in the moulded parts [85].

#### 2.1.4. Mould Temperature

Mould temperature is known as the temperature of the mould that needs to be controlled in order to solidify the molten plastic material that flows into the mould cavities towards the ejection temperature. Previous studies showed that mould temperature is one of the significant processing parameters that affects warpage and shrinkage defects [83,87]. Kamaruddin et al. [86] examined mould temperature using the Taguchi methods, and reported that the shrinkage of moulded parts affected by mould temperature is a critical factor. This supports the findings of a study by Chen et al. [89] which found that the temperature of the mould plays a role in the shrinkage of the resulting moulded products in both the transverse and longitudinal axes. In addition, mould temperature cannot be set directly but it can be controlled by controlling the temperature of coolant used in the injection moulding process.

#### 2.1.5. Packing Time

The packing time is known as the time required to fill the mould cavities without pressing the mould or flashing the finished parts entirely with additional material [90]. The packing time is generally determined by the freeze time of the gate [91]. When gates freeze, the material is not permitted to flow into the mould cavities. Nevertheless, if the packing time is shorter, the molten material returns to the feeding system and causes a backflow phenomenon [89,92].

It can be seen that, in terms of material used as mould inserts for injection moulding, the thermal conductivity (which influences the melt temperature, mould temperature, packing time, and definitely cooling time) and compressive strength (which influences packing pressure and reliability of mould inserts) are important parameters that require the attention of the mould fabrication industries.

### 2.2. Mould Base Material

The selection of material for mould base parts depends on the product that needs to be manufactured. Choosing suitable materials can help a company to save costs and time. The materials of the mould base are divided into four types, which are mild steel, high-alloy steel, stainless steel, and tool steel, as tabulated in Table 2 [83,84,85,86,87].

Mild steel is a type of iron that has varied levels of carbon added to it and no addition of other elements. There are different percentages of carbon where the carbon content ranges from mild, to medium, to high. Examples of carbon steel are carbon steel 1018 and 1050 [83,86]. High-alloy steel is a variety of steel that is alloyed with additional components ranging from 1 wt. % to 50 wt. % through the addition of carbon to enhance the material’s different qualities.

High-alloy steel is therefore made of iron that has been alloyed with additional elements including copper, chromium, and aluminium. It can also alloy more than two metals. Examples of alloy steel are alloy steel AISI 4130 and AISI M2 [35]. Stainless steel provides excellent corrosion resistance and machinability. Stainless steel is a class of iron-based alloys notable for their corrosion and heat resistance.

Furthermore, stainless steel is produced by adding chromium at a rate of about 11% and the use of stainless steel is selected because it does not corrode or oxidise. Stainless steel does not require stress relief because its material qualities are stable. Examples of stainless steel are stainless steel 420, 316L, and 17-4 PH [88,92,93]. Tool steel refers to a range of carbon and alloy steels that are especially well-suited to be produced into tools.

In addition, tool steel contains elements such as tungsten, vanadium, cobalt, and molybdenum [94]. These elements are used to improve hardenability and generate harder and more thermally stable carbides. Examples of tool steels are tool steel O-1, A-6, S-7, D-2, P-20, and H13 [83,88]. RT is the AM technology that refers to the manufacturing methods of tooling [94,95,96].

Injection mould bases can be made from a wide variety of materials. However, selecting the right mould base material is essential for making high-quality components, since different materials have different properties.

#### Selecting Mould Base Material

Material selection for the mould base is important because it will affect the performance of the mould. Selecting the suitable material during the tool-making stage can reduce cost. Several factors need to be considered, which are strength, good wear resistance, excellent surface finish, dimensional stability, machinability, and corrosion resistance. First, highly compressive loads must be able to be absorbed by the material without cracking or splitting. Next, good wear resistance is needed so that the mould can be used longer. Good surface finish is also vital to be considered because it will affect the product surface. Other parameters also need to be considered so that the product can be used longer, and to save cost and time. An example of this consideration is the use of H13 which is selected because it can perform well at high temperatures, and has high dimensional stability, hardness, and wear resistance [97]. The recommended mould material for transparent products is stainless steel AISI 420, which has a hardness of up to 54HRC [98].

On the other hand, mould inserts are assembled in a mould base and form the cavities where the molten plastic will be injected to form the products. Therefore, the material of the mould insert is an important aspect that will have a direct impact on the defects of the moulded parts produced.

### 2.3. Mould Insert Material

The material of a mould insert will affect the cooling time of a product as it influences the overall cycle time of the injection moulding process [36]. Other than that, improving cooling time can also reduce defects such as shrinkage and warpage [90,99,100]. Tool steel material takes longer to achieve the ejection temperature than pure copper (Cu) and beryllium copper (BeCu) as tabulated in Table 3 [86]. This is because Cu and BeCu have higher thermal conductivities which can remove more heat than tool steel material. It is important because the temperature needs to be evenly distributed from the cavity to the core of the mould [84]. Although pure copper is proven to be the best according to simulation results, other factors need to be considered in choosing the mould insert material, including properties such as hardness. The hardness of BeCu is higher compared to pure copper and other properties that need to be considered are, namely, durability and resistance to non-oxidising acids.

However, the materials used to fabricate mould inserts for the product designed in the development stage do not have to be the same as materials used for the hard tooling (mould used for mass production) because the product design is not yet finalised and there are still some tests and evaluations to be carried out, as well as a need to improve the product’s features in terms of ease of assembly and reliability tests in order to ensure the high quality of product. An alternative material of mould inserts for low production in the product development industry is in high demand, especially in the effort to reduce the expenses in the research and development stage.

#### 2.3.1. Alternative Materials for the Mould Insert

Small numbers of functional plastic parts that range from five to one thousand units are usually needed during the product development stage to confirm the development stage before mass manufacturing. An alternative material is required for mould inserts to reduce the cost, time, part quality, and production number [48]. Currently, the alternative material that is used in mould insert fabrication is epoxy resin. The different types of epoxy resin with their properties are listed in Table 4 [101].

Nevertheless, there are some restrictions when using epoxy as a mould insert in RT for injection moulding. Epoxy has limitations that must be overcome, such as its low hardness and strength [103]. Geopolymer can be used to replace epoxy since it is robust and strong and is now utilised in building concrete. In addition, it preserves the environment, reduces cost, and supports sustainability of waste management systems [81,94,95,104,105]. As an implication, industries related to mould-making will benefit the most due to the reduced cost when using recycled materials.

#### 2.3.2. Rapid Tooling (RT) Mould Inserts

Rapid tooling is an example of how rapid prototyping is used in the manufacturing industry. It enables the rapid and low-cost construction of moulds for small batches of manufacturing goods. Tooling may be either harsh or soft, and can be classified as direct or indirect [9]. An efficient method of direct tooling involves the use of soft materials in a rapid prototype process such as stereolithography material [96,106,107,108]. Numerous different tools, such those made of powder metal [96,103], are made of tough materials, and in the indirect tooling method, a casting pattern is made by rapid prototyping and then used to manufacture the proper tool. Due to its simplicity of usage in producing mould inserts, aluminium-filled epoxy resin [109,110] is becoming a popular soft material. Silicone rubber is mostly utilised in the manufacture of indirect tools [107].

The most significant factor for injection moulds made utilising the RT process is tool life. RP technology has improved to the point where tools directly generated by RP machines should represent all of the model’s various elements and features accurately and precisely. On the other hand, many soft rapid prototyping materials are unable to tolerate sufficiently high pressure and melt temperature owing to their poor heat conductivity [107], resulting in shortened useful life of the instrument. Although other methods, including metal laser sintering, may be employed to apply metal coatings on pliable materials [108] to enhance their hardness, it will raise the manufacturing process’s difficulties. Alternatively, epoxy resin is often used in indirect moulds due to its plasticity or compatibility with casting models. The use of metal powder may greatly boost its hardness and heat conductivity, prolonging tool life even more. However, this does not prevent the epoxy resin from hardening within the mould chamber and becoming brittle. Indirectly crafted tools thus wear very quickly [104]. Some of the studies concentrating on RT are listed in Table 5.

**Figure 5:**
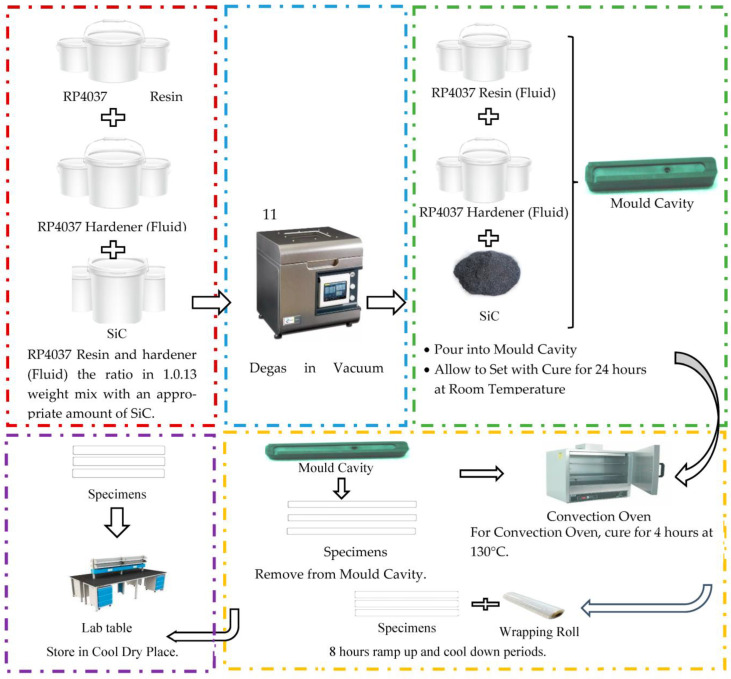
Method for setting up a tooling board.

Tomori et al. [110] investigated how changing the material formulation and determining the validity of composite tooling boards affected mould efficiency and component quality. An example of the method for setting up a tooling board is illustrated in Figure 5. The boards were constructed using three materials: RP4037 (fluid), RP4037 hardener, and silicon carbide (SiC) filler (powder). For the six moulds, two cutting speeds (1.00 and 1.66 m/s) and three tooling board formulations (28.5%, 34.75%, and 39.9% wt. % SiC filler) were used. The surface roughness of the moulded components served as the study’s response variable, while cutting speed served as the study’s independent parameter. As there was no visible mould damage, the physical structure of the mould was unchanged by SiC concentration and cutting speed. This discovery indicated that the SiC content in the mould has a significant impact on the surface roughness of the moulded items. Additionally, the flexural strength rose with the SiC filler concentration (from 58.75 to 66.49 MPa), following a pattern comparable to the heat conductivity of the mould material. The influence of filler concentration primarily on the direction of welding for moulded components was not examined in this research.

Senthilkumar et al. [111] studied the effects of epoxy resin on the mechanical characteristics of aluminium (Al) particles. The sample was cast utilising Al filler mixed into epoxy resin at various concentrations. Optical microscopy revealed that the Al particles were uniformly dispersed throughout the epoxy resin matrix. These results show that increasing the amount of Al particles inside the epoxy resin matrix significantly raises both the thermal conductivity (3.97 to 5.39 W/mK) and the hardness value of the composite (69 to 89 RHL). The sample’s fatigue life decreased from 15,786 cycles to 734 cycles as the Al content of the epoxy resin increased. The best percentage of Al filler particle for enhancing mould performance and durability was found to be between 45 and 55 wt.% There was an improvement of 72 RHL in durability, 10,011 cycles in fatigue resistance, and 4.06 W/mK in thermal conductivity. However, the hardness value increased by 4.34% for every 5% increase in Al filler particles, which might reduce the fatigue life by 36.58%. Nevertheless, there has been no further research on the moulded components’ flexural strength, compressive strength, tensile strength, or surface appearance.

Srivastava and Verma [27] attempted to determine how the addition of Cu and Al particles to epoxy resin composites altered their mechanical properties. Epoxy resin was mixed with Cu and Al particles (1, 5, 8, and 10 wt. %) to create a variety of filler compositions. The results of the mechanical tests showed that the epoxy resin with Al reinforcement has excellent tensile properties, with a tensile strength of 104.5 MPa at 1 wt. %, while the epoxy resin composites with Cu filler was optimal in the hardness test (22.4 kgF/mm^2^ at 8 wt. %) and had a compressive strength of 65 MPa at 10 wt. %. In addition, epoxy resin composites filled with Cu demonstrated better performance than those filled with Al despite having a lower hardness. This finding demonstrated that the tensile strength, wear loss, and hardness of the material all decreased steadily with increasing filler content, whereas the compressive strength, friction coefficient, and hardness all showed an increase. However, the impact of the welding direction on the surface of the moulded components is yet to be determined.

Fernandes et al. [26] studied the dimensions and mechanical characteristics of epoxy resin/Al insert-moulded PP injection components for RT. A 140 mm diameter sphere was made up of five chambers with 2 mm thick walls that formed the work’s central geometrical component. The length of the test was 60 mm, the diameter of the entrance was 6.5 mm, and the draught angle was 2°. To test the suggested mould, a novel hybrid mould comprising epoxy resin and Al was employed in this work to insert polypropylene (PP) pieces. In addition, comparable pieces were inserted to use an AISI P20 (conventional) steel mould, the same as in the genuine application. Epoxy resin/Al insert-filled components had slightly higher tensile strength at yield (22.0 ± 5.0 MPa) than steel AISI P20 insert-filled components (20.0 ± 4.5 MPa), but the difference was not statistically significant. Epoxy resin/Al-injected parts had lower values for ultimate tensile strength, elongation at break, and modulus of elasticity than steel AISI P20-injected parts. Furthermore, the Shore D hardness of objects formed by AISI P20 steel inserts increased by 8.5% in comparison to goods moulded by epoxy/Al inserts. When compared to components injected using an epoxy/Al mould, those injected using an AISI P20 steel mould showed less shrinkage. Based on these findings, epoxy/Al moulding blocks may be a high-quality alternative to fast tooling for producing single units or small series. Furthermore, this research did not investigate whether the orientation of welding on the moulded components was affected by the impact.

Khushairi et al. [112] investigated various epoxy compositions using Al, Cu, and brass fillers which were tested for their mechanical and thermal properties. In Al-filled epoxy, different combinations of brass and Cu filler (10, 20, and 30% wt. %) were used. Brass and Cu densities were 2.22 g/cm^3^ and 2.08 g/cm^3^ at the optimum filler content, respectively. When 30% Cu fillers were added to an epoxy matrix, the total thermal diffusivity (1.12 mm^2^/s) and thermal conductivity (1.87 W/mK) were the maximum, but adding brass had no effect on thermal properties. When 20% brass filler was added, compressive strength increased from 76.8 MPa to 93.2 MPa, whereas 10% Cu filler raised compressive strength from 76.8 MPa to 80.8 MPa. As a result of porosity, multiple metal fillers diminished the compressive strength. According to this research, fillers boost mechanical, thermal, and density properties of Al-filled epoxy. Nonetheless, a careful evaluation of the surface characteristics, notably the welding line of the moulded components, is necessary to determine the moulded parts’ quality.

Kuo and Lin [113] examined the quick injection moulding of Fresnel lenses from liquid silicone rubber. The experiment was conducted utilising RT and liquid silicone rubber (LSR) parts to build a horizontal LSR moulding machine (Allrounder 370S 700–290, ARBURG, Loßburg, Germany). Injection moulds for LSR injection moulding could be manufactured using Al-filled epoxy resin. The total microgroove depth and width of the Al-filled epoxy resin mould were 90.5% and 98.9%, respectively. LSR-moulded components exhibited typical microgroove depth and width transcription rates of roughly 91.5% and 99.2%, respectively. LSR-moulded components’ microgroove depth as well as width may be modified to within 1 m. The mean surface polish of the Al-filled epoxy resins increased by around 12.5 nm following 200 LSR injector test cycles. However, further testing on tensile strength, compressive strength, hardness, and density, as well as weld line observations, is essential to understand the impact of quick injection moulding on the recommended mould in terms of moulded component quality.

From the review that has been carried out, it can be seen that numerous elements such as flexural strength, hardness, thermal conductivity, tensile strength, compressive strength, density, thermal diffusivity, and surface roughness of the new material introduced are important factors that need to be considered prior to its use as mould inserts for RT in the injection moulding process.

### 2.4. Geopolymer

A geopolymer is formed by combining a dry solid containing high aluminosilicate content, called a precursor, with alkaline solution and other ingredients if needed [114]. It is a semicrystalline, three-dimensional structure made of the tetrahedral structures of silica and alumina that share oxygen [115]. Geopolymer precursor can be obtained in two ways: from geological origin or industrial by-products. Examples of geological origins are kaolinite and clay, while industrial by-products are fly ash (FA), wheat straw ash, and furnace ash. Geopolymers are activated using high-alkali solution for the polymeric reaction to occur by using sodium hydroxide (NaOH), potassium hydroxide (KOH), or a mixture of sodium oxide (N_2_O) and silicon monoxide (SiO) [116].

The geopolymer concrete curing process has a significant impact on mechanical characteristics and microstructure development [117,118]. Excellent mechanical strength, reduced creep, improved acid resistance, and minimal danger of shrinkage are all characteristics of geopolymer concrete [41,119,120]. The durability of waste pozzolan-based geopolymer concrete that is cured at high temperatures has been extensively studied [121,122,123,124]. By curing the geopolymer at a higher temperature, one may enhance the geopolymer’s mechanical properties, polymerisation level, microstructure density, and overall strength [117,125,126,127].

Geopolymers come in a variety of unique shapes, and each type has certain properties. Geopolymers are an alternative material in the tooling industry. However, changing the geopolymer composition will change the qualities of the geopolymer, where selecting the correct geopolymer precursor will give the tooling industry greater advantages.

#### 2.4.1. Effect of Different Geopolymer Precursors on Mechanical Properties of Geopolymer

Concrete for building uses geopolymer because of its great compressive strength. Its mechanical qualities, however, can vary depending on the type of geopolymer used [128,129,130,131]. Previous studies employing various geopolymer precursors are presented in Table 6.

**Figure 6:**
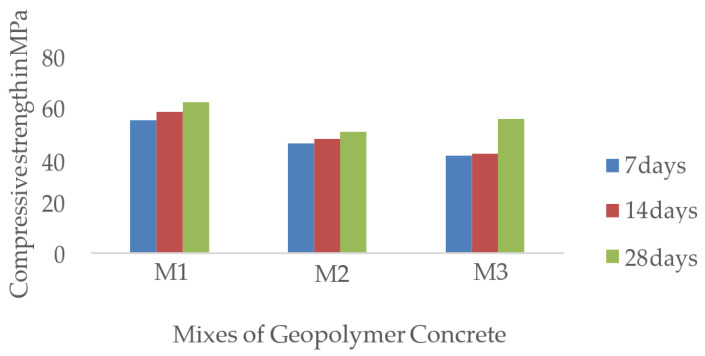
Compressive strength of geopolymer concrete with different mixture compositions [131].

Girish et al. [131] investigated the feasibility of employing geopolymer concrete as fine aggregate in stiff paving-grade concrete comprising quarry dust and sand. The 60/40 mixture consisted of fly ash and ground granulated blast furnace slag (GGBS), had different solid–liquid ratios, and was examined at 3, 14, and 28 days. Increasing the molar ratio of the NaOH solution from 8M to 14M increased the strength of the resulting concrete but reduced the solution’s workability. The experiment used a 12M NaOH solution, and the fine aggregates included both quarry dust and sand. The maximum strength was 62.15 MPa, and it was reached after 28 days. The results of the compressive strength test as depicted in Figure 6 showed that the strength of all the mixtures had increased. The achieved compressive strength at 28 days was more than the 40 MPa minimum required for stiff pavement cement concrete. However, research needs to be undertaken to investigate whether the compressive strength of geopolymer concrete is affected by the substitution of quarry dust for sand.

Girish et al. [132] investigated self-consolidating geopolymer concrete for fixed-form pavement. Optimal strength geopolymer concrete is produced with a SiO_2_/Al_2_O_3_ ratio between 3.0 and 3.8 and a Na_2_O/Al_2_O_3_ ratio of 1. Compressive strength of 40 MPa was targeted for this mixture, which also included class F fly ash, ground blast furnace slag (GGBS), NaOH particles and solution form (molar concentration: 10 and 12), Na_2_SiO_3_ (A-53 grade), fine aggregate (quarry dust and river sand), coarse aggregate (below −20 mm), retarder (Conplast SP500), sugar solution, and water. The average compressive strength of the ambient-cured M10 mix after 28 days was 56.47 MPa, which is 40% higher than the intended compressive strength. At day 56, the compressive strength had increased to a peak of 71.78 MPa. However, as highlighted in Table 7, the proposed combination lacks considerable green strength, which is essential for slip-form paving applications, due to its low viscosity and yield stress. To make the SGC more environmentally friendly and appropriate for sliding mould applications, it might be beneficial to include nanoclays and/or fibres in the material.

**Figure 7:**
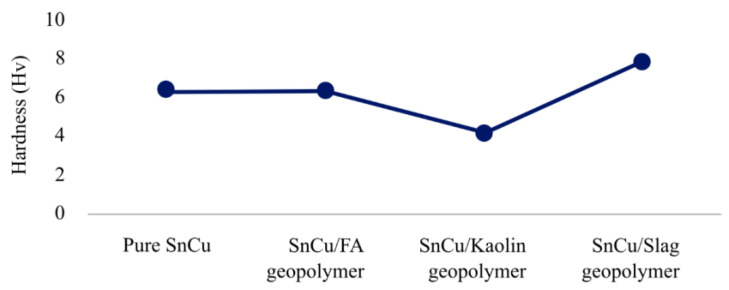
Different compositions of composite solder hardness value [133].

Izzati et al. [133] evaluated the use of different levels of geopolymer. No geopolymers, 1.0 wt. % fly ash, kaolin, or slag geopolymer particles were added to Sn-0.7Cu. All the mix designs were cured for 3 days and the temperature of curing for fly ash and slag was 27 °C and that for kaolin was 80 °C. As illustrated in Figure 7, using slag geopolymer is more challenging compared to not using geopolymer and using other geopolymers. Future research can attempt at using a higher percentage of geopolymer to test the composition’s hardness. This may result in higher hardness compared to 1% geopolymer. To be comparable to other geopolymers, future research needs improve its preparation procedure in terms of curing temperature.

**Figure 8:**
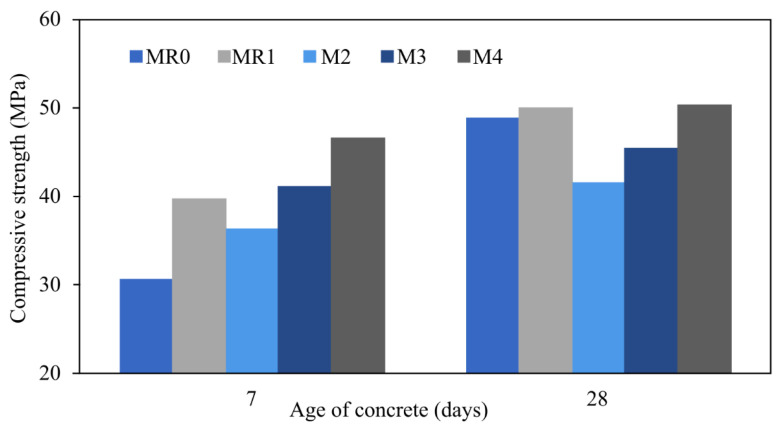
Compressive strength of geopolymer with different mixtures [134].

Hussein and Fawzi [134] tested various geopolymer contents in mix composition. The normal composition was cement with fine aggregate and coarse aggregate and 0% and 5% copper fibre, while the geopolymer composition had varied amounts of fly ash (FA) and slag with fine aggregate and coarse aggregate and 0% and 5% copper fibre. The preparation was cured at 40 °C for seven to twenty-eight days to evaluate compressive strength, splitting tensile strength, and bending strength. Figure 8 demonstrates that the maximum compressive strength, splitting tensile strength, and bending strength increase when the FA to ground granulated blast furnace slag (GGBFS) ratio is 0.55:0.45 with 0.5% copper wire fibre. It indicates that the compressive strength increases as the GGBFS level rises. The maximum strength of the geopolymer content can be determined by employing longer curing times and greater FA to GGBFS ratios.

**Figure 9:**
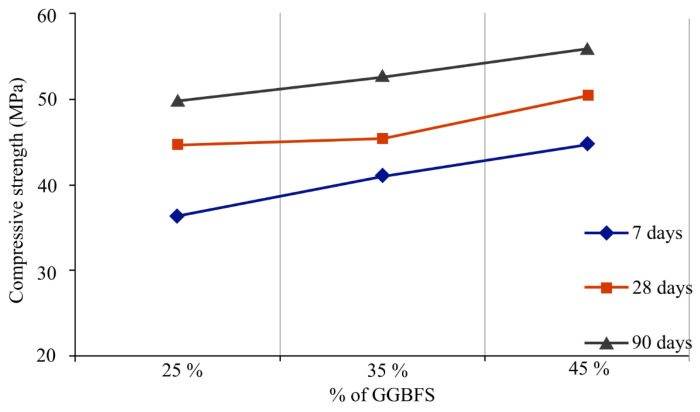
Different content percentages of GGBFS show different compressive strengths [135].

Hussein and Fawzi [135] analysed different contents of geopolymer by using different ratios of fly ash (FA) to ground granulated blast furnace slag (GGBFS). Cement, fine aggregate, and coarse aggregate were used in the preparation of MR0 and MR1, while fly ash to slag ratios for MG0, MG1, MG2, and MG3 were 0.75:0.25, 0.65:0.35, and 0.55:0.45 and mixed with fine aggregate and coarse aggregate in MR1, MG1, MG2, and MG3 with 0.5% copper fibre added. The preparation was cured at 40 °C for seven and twenty-eight days. As depicted in Figure 9, the larger the proportion of GGBFS, the greater the compressive strength and, at ninety days, 45% GGBFS had the highest compressive strength. MG3 with a content of 45% GGBFS shows the highest split tensile strength and flexural strength. To determine the ideal fly ash to slag ratio for assessing hardness, an analysis with a higher fly ash to slag ratio could be carried out.

According to the review, mechanical qualities can be improved by utilising slag geopolymer. Research is necessary to determine whether a particular geopolymer can enhance mechanical properties. Furthermore, according to the studies mentioned, there are several preparations that would affect the strength, therefore the sample preparation procedure should be fixed, such as curing at the same temperature, to ensure that the results are unaffected. Varied drying times will result in different compressive strengths.

The mechanical characteristics of geosynthetics are affected by several geosynthetic precursors. The strength of geosynthetic polymers is improved by using varied ratios of sodium silicate/sodium hydroxide and fly ash/alkaline activators.

#### 2.4.2. Effect of Different Ratios of Sodium Silicate/Sodium Hydroxide and Fly Ash/Alkaline Activators on the Mechanical Properties of Geopolymer

The current investigation looks into the influence of sodium silicate/sodium hydroxide ratios on geopolymer feasibility. Different studies showing the various proportions of sodium silicate/sodium hydroxide and fly ash/alkaline activator to improve geopolymer properties are listed in Table 8 [27,111]. The ideal preparation of fly ash can be determined by testing varying concentrations of sodium silicate, sodium hydroxide, fly ash, and alkaline activator.

Morsy et al. [136] evaluated the influence of sodium silicate/sodium hydroxide ratios on the viability of fly ash-based geopolymer synthesis at 80 °C. In this study, 10 M NaOH was combined with Na_2_SiO_3_ and alkaline activator ratios of 0.5, 1.0, 1.5, 2.0, and 2.5. The compressive strength of fly ash geopolymer mortars increased with age at 3, 7, 28, and 60 days. The compressive strengths of fly ash geopolymer mortars M1, M2, M3, M4, and M5 after three days were 34.7, 61.6, 40.4, 40.5, and 22.3 MPa, respectively. The S/N ratio of alkali activator had a significant impact on the strength of low-calcium fly ash geopolymer cured at 80 °C. Maximum strength was achieved when the ratio of sodium silicate to sodium hydroxide (S/N) was equal to 1. Other than that, future research should investigate preparation methods for mixtures and ensuring homogeneity so that they are comparable with other geopolymers.

According to Liyana et al. [137], in their study, the proportions of Na_2_SiO_3_/NaOH solution and fly ash to alkaline activator were synthesised in four different ratios: 1.0, 1.5, 2.0, and 2.5, in a 24 h period during which curing was carried out at room temperature. According to the results, the fly ash/alkaline activator ratio of 2.0 had the highest results compared to other ratios, and the sodium silicate/sodium hydroxide ratio of 2.5 had the highest results compared to other ratios. The best mechanical properties can be obtained through research using various molarities and curing temperatures.

The study by Bakri et al. [138] used a 12 M concentration of NaOH and fly ash to alkaline activator ratios of 0.5, 1.0, 1.5, 2.0, 2.5, and 3.0. Only the three ratios of 1.5, 2.0, and 2.5 were employed. Due to the geopolymer paste’s high workability, which makes it challenging to handle, the ratios of 0.5 and 1.0 could not be used, and the ratio 3.0 could not be used due to the paste’s low workability. Five different ratios of Na_2_SiO_3_/NaOH were used: 0.5, 1.0, 1.5, 2.0, and 2.5. The sample was cured for 24 h at 70 °C before being tested for compressive strength on the seventh day. The fly ash/alkaline activator ratio of 2.0 and the sodium silicate/sodium hydroxide ratio of 2.5 had the maximum compressive strength. Future studies could examine various curing temperatures to achieve the best compressive strength.

**Figure 10:**
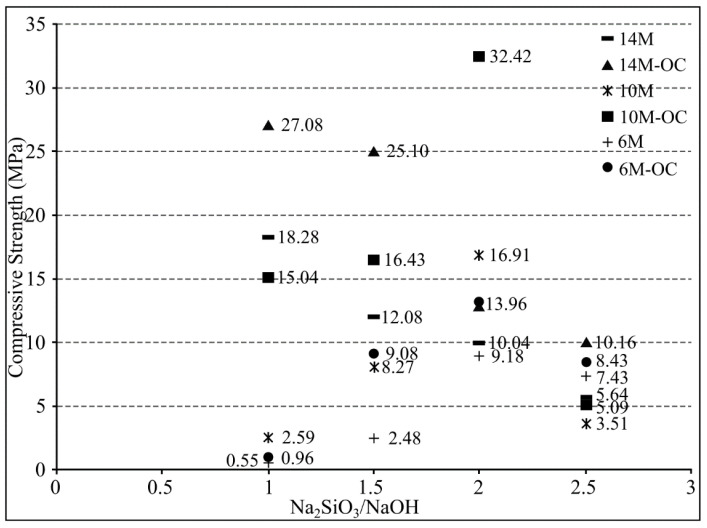
Compressive strengths of alkali-activated fly ash/slag specimens based on alkaline activator ratio type [139].

Nis [139] investigated geopolymer content using various NaOH concentrations and sodium silicate to sodium hydroxide ratios. The sodium silicate (Na_2_SiO_3_) and sodium hydroxide (NaOH) solutions were used with four different sodium silicate to sodium hydroxide ratios (1, 1.5, 2, and 2.5) and three different molarities (6 M, 10 M, and 14 M) for alkali activation to evaluate the impact of these parameters on the compressive strength of the alkali-activated fly ash/slag concrete under ambient-curing (AC) and delayed oven-curing (OC) conditions. The specimens’ compressive strengths varied greatly with molarity concentration; those with the greatest NaOH molarity (14 M) concentration had the greatest compressive strength, as depicted in Figure 10. Other than that, more research can consider investigating the impact of oven-curing conditions on compressive strength.

Abdullah et al. [140] investigated several curing temperatures with a constant NaOH concentration of 12 M using different fly ash/alkaline activator ratios and Na_2_SiO_3_/NaOH ratios. The samples were cured at different temperatures from 40 °C to 80 °C for 24 h and compressive strength was tested on the seventh day. The fly ash/alkaline activator ratio of 2.0, sodium silicate/sodium hydroxide ratio of 2.5, and curing temperature of 60 °C resulted in the maximum compressive strength. Different curing days may be investigated in order to enhance compressive strength.

Based on various studies [137,138,139,140,141], the mechanical characteristics may be affected by the use of different ratios of sodium silicate, sodium hydroxide, and fly ash/alkaline activator. The strongest strength resulted from the fly ash/alkaline activator ratio of 2.0 and the sodium silicate/sodium hydroxide ratio of 2.5, which were used as the ideal ratio for sample preparation. However, from the previous investigation, more improvements can be made, which are optimising the curing temperature and curing durations as using various curing durations can potentially increase the geopolymer’s mechanical qualities.

Although the mechanical characteristics are influenced by the ratio of sodium silicate/sodium hydroxide and fly ash/alkaline activator, the preparation of different molarities of sodium hydroxide is another key aspect that influences overall mechanical characteristics of geopolymer.

#### 2.4.3. Effect of Sodium Hydroxide Molarity on the Mechanical Properties of Geopolymer

The molarity of the alkali activator, the curing temperature, the number of days, and other parameters all have an impact on the sample’s characteristics during the creation of the geopolymer [27,111,112,113]. The different molarities of sodium hydroxide, that acts as an alkali activator and affects the mechanical properties of geopolymer, are listed in Table 9.

**Figure 11:**
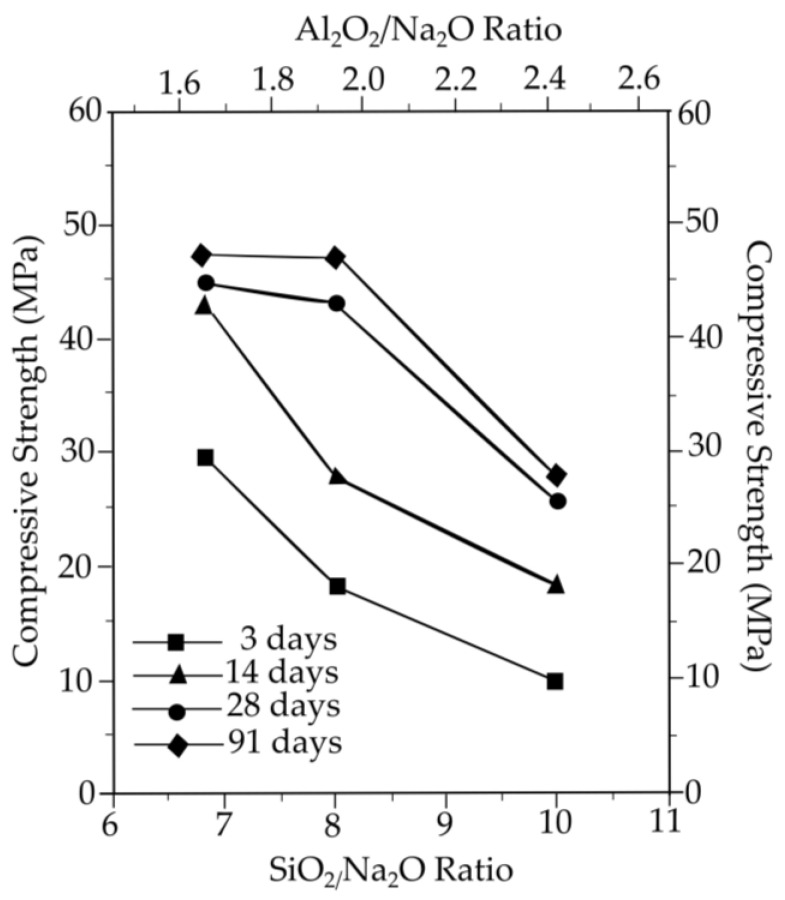
Compressive strength versus ages for molarity of NaOH [143].

Gum et al. [143] studied the impact of making geopolymer concrete with an alkaline activator on the compressive strength of mortars using fly ash as a binder and different curing temperatures and moles of sodium hydroxide. Fly ash was combined with a mixture of 6, 9, and 12 M NaOH, and the curing conditions were 60 °C in the oven and 20 °C outside for 7 classes of curing days. After the chemicals were mixed, it was poured into moulds with dimensions of 50 mm × 50 mm × 50 mm and measured for compressive strength according to ASTM C 109. An alkaline activator that used NaOH at a higher molarity demonstrated increased compressive strength. The compressive strength decreased as the SiO_2_/Na_2_O and Al_2_O_3_/Na_2_O ratios increased. When the SiO_2_/Na_2_O ratio exceeded 8.01 and the Al_2_O_3_/Na_2_O ratio exceeded 1.94, the strength decrease rate appeared to accelerate sharply at 28 days. Based on these findings, the strength at 28 days for series 1 appeared to have increased by more than 1.7 times at a NaOH molarity of 9 M when compared to a molarity of 6 M. However, the 9 M and 12 M results showed nearly identical strengths. This highlights the significance of the SiO_2_/Al_2_O_3_, SiO_2_/Na_2_O, and Al_2_O_3_/Na_2_O ratios in alkali-activated geopolymer based on fly ash. As SiO_2_/Al_2_O_3_ was constant in this investigation, the values of 8.01 and 1.94 for SiO_2_/Na_2_O and Al_2_O_2_/Na_2_O ratios yielded the best strength development. The use of NaOH and sodium silicate (SiO_2_/Na_2_O = 8) in a 1:1 ratio demonstrated that it is possible to activate the geopolymerisation of fly ash and create a significant increase in strength, with a compressive strength of around 47 MPa. The evaluations of the impacts of the SiO_2_/Na_2_O and Al_2_O_3_/Na_2_O ratios on strength under equal SiO_2_/Al_2_O_3_ ratios are illustrated in Figure 11. The requirement for high-strength concrete is over 40 MPa, demonstrating the possibility of employing fly ash as a cement substitute. Future research can evaluate whether increasing the molarity and pH of NaOH during the curing process will increase compressive strength, including multiple curing temperatures.

**Figure 12:**
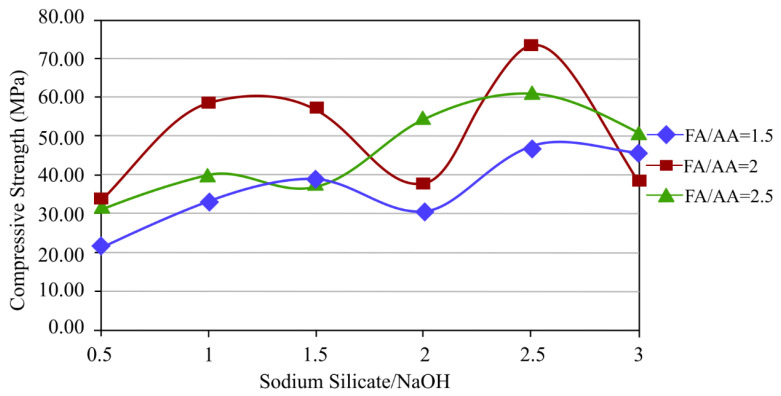
Time versus NaOH solution molarity [144].

Lee et al. [144] analysed the effects of increasing amounts of slag, water glass, and varying curing temperatures and NaOH molarities on curing time reduction. In the preparation, the alkali activators were water glass (Korean Industrial Standards, KS 3-grade; SiO_2_ (29%), Na_2_O (10%), H_2_O (61%, specific gravity 1.38 g/mL), and 98% pure NaOH. The room temperature for the combined alkali-activated fly ash/slag paste was between 17 °C and 28 °C. For setting time tests, the molarity of NaOH was 4 M and 6 M, and the mass ratio of NaOH was 0.5, 1.0, and 1.5. Then, 8 M NaOH was used to accelerate the setting of alkali-activated fly ash/slag paste. For each mixed sample, a 100 mm × 200 mm cylinder mould was employed. The compressive strength and setting times of ASTM C 191-08 [139] were evaluated at 3, 7, 14, 28, and 56 days of curing. At 17 °C, the alkali-activated fly ash/slag paste took 55 min to start and 160 min to finish when the NaOH solution was 4 M and the water glass to NaOH solution by weight ratio was 0.5, as illustrated in Figure 12. Due to the presence of slag and water glass, the molarity of NaOH rose while the alkali activator’s duration shortened. The quantity of slag grew by 25% and 30% after 28 days, respectively, but reduced after 56 days due to crack growth. Future research can examine different NaOH molarities to determine whether they can boost compressive strength.

**Figure 13:**
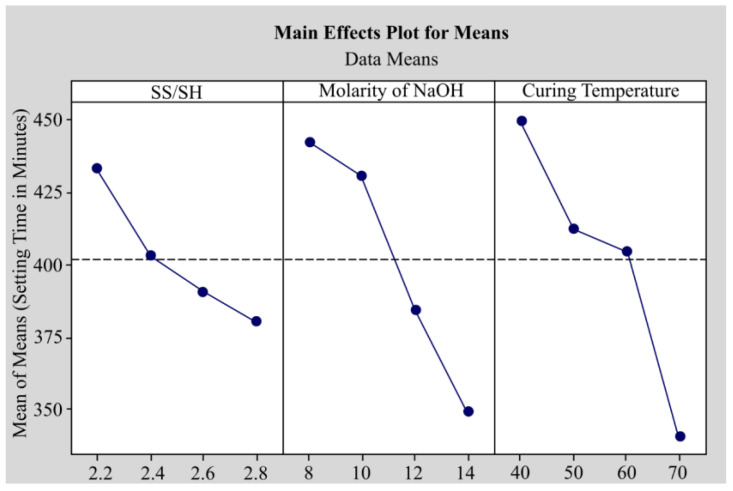
Main effects plot for S/N ratios based on compressive strength [146].

Khan et al. [146] examined the material properties of fly ash, copper slag, and crusher dust at different curing temperatures and NaOH concentrations. There were 16 different mix designs that used varying curing temperatures and NaOH concentrations. The design was cured for 28 days before testing, and the analysis revealed that the sodium silicate/sodium hydroxide (SS/SH) ratio should be maintained at 2.4. The molarity of NaOH should be kept at 14 M to produce maximum strength and dotted line was an average region, as shown in Figure 13. The setting time was found to decrease from 449.8 min to 340.8 min. There are some limitations, such as the fact that the greater the molarity, the greater the compressive strength, and this could be due to secondary parameters that may affect the performance of geopolymer, including mixing time and other parameters that can influence the complexity of the mix design; therefore, additional research is required to determine their characteristics.

**Figure 14:**
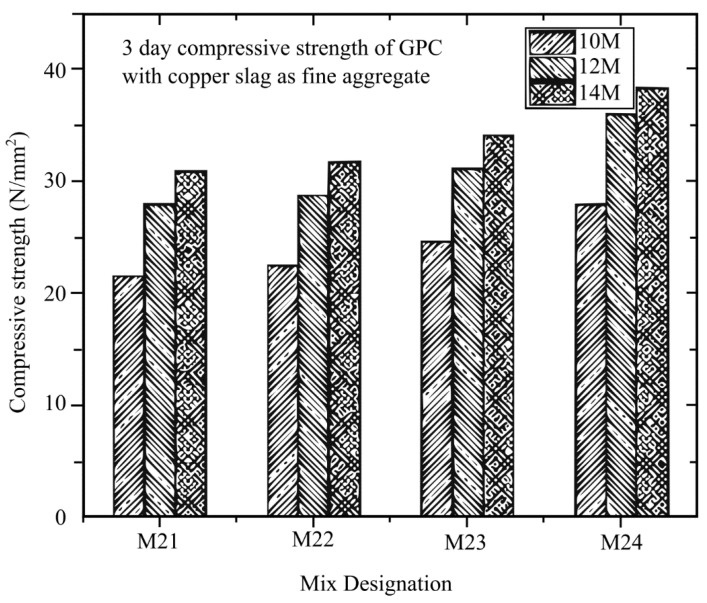
Different molarities in the mix design of GPC and copper slag at 3 days [145].

**Figure 15:**
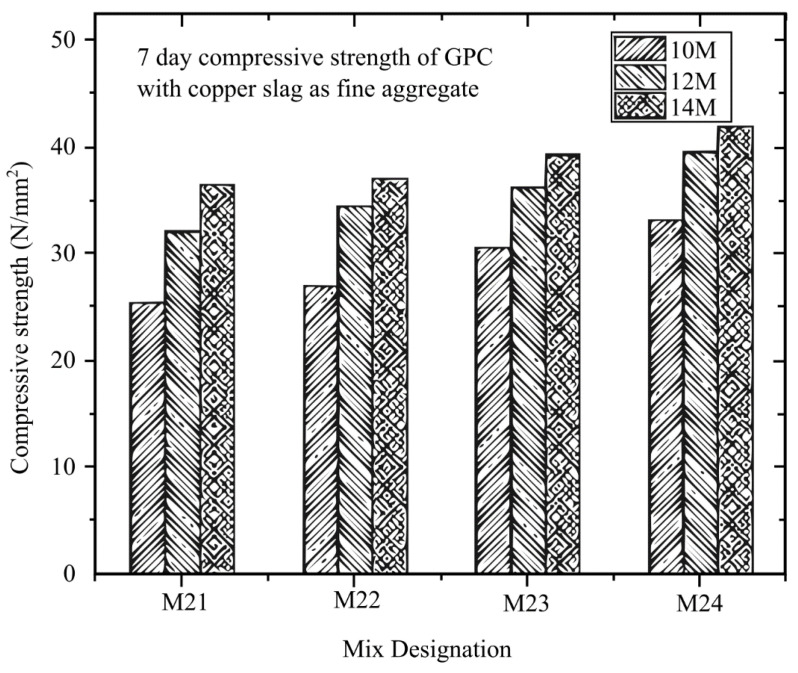
Different molarities in the mix design of GPC and copper slag at 7 days [145].

**Figure 16:**
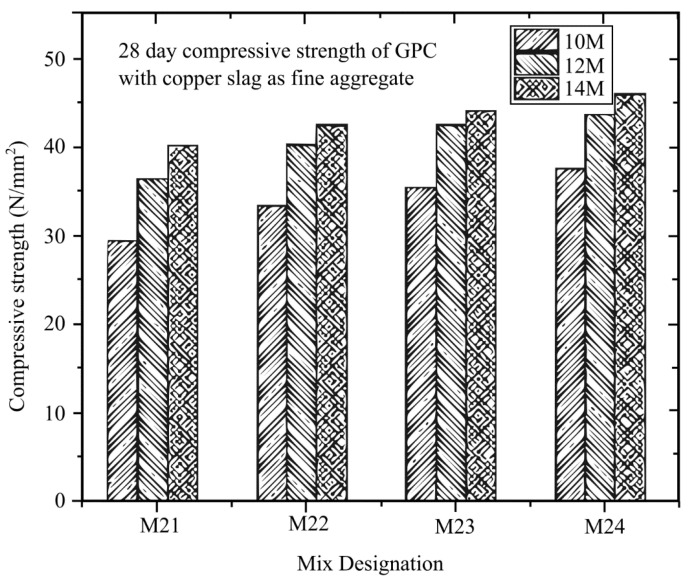
Different molarities in the mix design of GPC and copper slag at 28 days [145].

Rathanasalam et al. [145] investigated different sodium hydroxide (NaOH) molarities of 10 M, 12 M, and 14 M and developed a mixture utilising 5%, 10%, and 15% ultrafine ground granulated blast furnace slag (UFGGBFS) replacing fly ash, with crushed stone or copper slag. After curing for 3, 7, and 28 days at 60 °C, the compressive strength was evaluated. The compressive strength of all mix designs was tested using a 150 mm × 150 mm × 150 mm cube. From the different types of design with different curing days depicted in Figure 14, Figure 15 and Figure 16, it can be concluded that all the mixtures with 14 M NaOH concentration have the maximum compressive strength. Future studies can look into using higher NaOH molarities to determine the ideal NaOH molarity to make the mix design with the maximum compressive strength.

**Figure 17:**
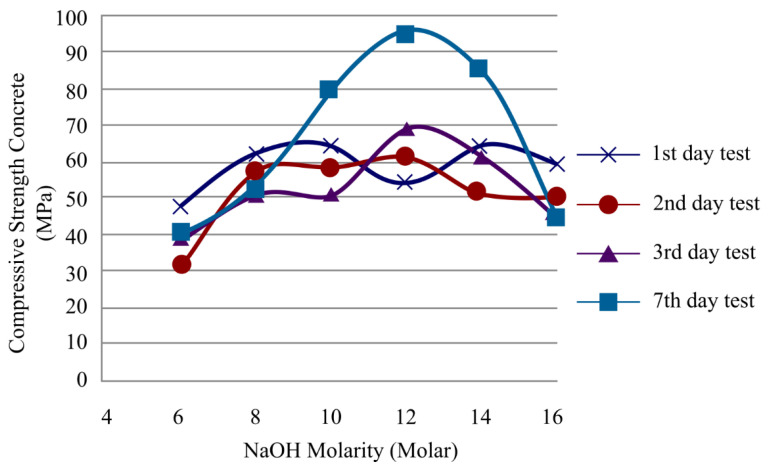
Compressive strength of all mixtures with different molarities and curing days [142].

Bakri et al. [142] investigated the compressive strength of fly ash at various sodium hydroxide molarities. The sodium hydroxide molarities of 6, 8, 10, 12, 14, and 16 M and 1, 2, 3, and 7 curing days were used for the mix design samples. The proportion of fly ash to alkali activator was maintained constant at 2.50, as was the proportion of sodium silicate to sodium hydroxide. Prior to testing, all mixtures were cured at 70 °C, and the results indicated that for sodium hydroxide with molarity of 12 M, the compressive strength result was the highest among the other molarities on the third day, and on the seventh day, it demonstrated the highest compressive strength, as illustrated in Figure 17. Future research can examine whether increasing the curing temperature will increase compressive strength.

Previous studies have investigated the effect of molarity of sodium hydroxide on mechanical properties. According to the majority of the studies, the higher the sodium hydroxide molarity, the higher the mechanical characteristics of the geopolymer. Although lower compressive strength is seen in mix designs when sodium hydroxide molarity is 15 M, according to research by Khan et al. [146], this may not be due to the influence of sodium hydroxide. First, it might be affected by the addition of other materials such as copper slag and crusher dust, as well as other aspects that lower compressive strength such as the SS/SH ratio and curing temperature. Although increasing the molarity improves the mechanical properties of the geopolymer, research by Fakhrabadi et al. [147] shows that when the sodium hydroxide molarity is 15 M, unconfined compressive strength is lower than when the sodium hydroxide molarity is 11 M, while research by Bakri et al. [142] suggested that molarity of 14 M is optimal for improving the mechanical properties of fly ash. The development of sodium aluminate silicate hydrate was caused by an increase in the molarity of sodium hydroxide (NASH) [148]. The use of sodium hydroxide with a high molarity may enhance the geopolymerisation reaction and the dissolution of initially solid materials, leading to better compressive strength [149].

The success of the geopolymer preparation demonstrates that it is possible to increase the material’s strength through geopolymer preparation. However, the low thermal conductivity of geopolymer can be improved by adding metal filler to the mould insert.

## 3. Summary and Future Works

A combination of RP technique with production tooling helps carry out RT more quickly but faces dimensional accuracy and surface finish issues. Moreover, the injection moulding process faces an issue with cooling time where most mould inserts fabricated using RP techniques have very low thermal conductivity, thus increasing the cooling time, which will definitely affect the cycle time to produce the parts.

Many researchers have started to explore the use of metal epoxy composite (MEC) as mould inserts for RT in the injection moulding process by using pure metal filler particles. However, epoxy that can withstand high temperatures (>220 °C) is hard to find and costly. Therefore, there is a potential opportunity for epoxy to be replaced by geopolymer materials, especially fly ash as raw material. Geopolymer material can withstand temperatures up to 1000 °C. Similarly, the compressive strength of epoxy is 68.95 MPa (10,000 psi) as compared to geopolymer which has strength of 60–80 MPa (8700–11,600 psi). The challenges of using geopolymer material are similar to those of epoxy resin in that optimal strength, good accuracy, acceptable surface finish, and good thermal characteristics must be determined. Based on the gaps found from the literature, recommendations for future studies are as follows:

i. The mechanical and metallurgical properties of GGMC mould inserts should be evaluated to provide significant information and benefits to mould-making and rapid tooling industries.
ii. The size precision and surface integrity of the GGMC mould inserts after the casting process should be evaluated accordingly and compared to the GGMC mould inserts after machining in order to produce precision plastic product with a high-quality surface finish.
iii. To enhance the qualities of the outcomes, various geopolymers filled with scrap metal fillers should be mixed to increase thermal conductivity, or two or more kinds of filler materials can be added to improve thermal conductivity.
iv. The purpose of carrying out RT before production tooling for mass production is to evaluate the part performance and mostly requires modification of the mould inserts. Thus, an investigation on the effects of dimensional accuracy and surface quality in the machining process is definitely required.

This review has provided a clear reference for future development of mould inserts for RT using GGMC material. Thus, initiative needs to be taken to conduct an analysis on the effect of incorporating metal particles in geopolymer material as mould inserts for RT and its relationship with compressive strength and thermal conductivity. Moreover, the integration of metal scraps from machining with geopolymer formed from waste makes this research more interesting. GGMC material should be examined for metallurgical parameters such as corrosion rate, coefficient of expansion, surface roughness, and additive manufacturability. Furthermore, the machinability and the reliability of GGMC mould inserts should be explored and evaluated accordingly. At the end of this research, the discovery of new sustainable green material will benefit moulding and rapid prototyping industries, including with its environmentally friendly attributes.

## Figures and Tables

**Table 1:** Six patents granted/published from 1990 to February 2023.

No.	Patent Number	Title	Inventor/s	Granted/Publication Date	Patent Summary
1	US4980242A	Fibre-reinforced metal composite	Tadashi Yamamoto, Michiyuki Suzuki, Yoshiharu Waku, Masahiro Tokuse [65]	25 December 1990	Aluminium matrix composite is a fibre-reinforced metal composite containing 6–11% nickel.
2	US6056915A	Rapid manufacture of metal and ceramic tooling	Mohammad Behi, Mike Zedalis, James M. Schoonover [66]	2 May 2000	Steel tooling is needed to produce near-net form, complex items in high volume. The technology is economical to make complex metal tooling for quick fabrication of complex shaped parts using conventional metal, ceramic, and plastic processes.
3	US6376098B1	Low-temperature, high-strength metal-matrix composite for rapid-prototyping and rapid-tooling	Furqan Zafar Shaikh, Howard Douglas Blair, Tsung-Yu Pan [67]	23 April 2002	Fibre to metal or alloy ratio can vary from 9:1 to 1:1. Fibres have an average diameter of 8 micrometres, and metal or alloy is distributed within them.
4	US20020187065A1	Method for the rapid fabrication of mould inserts	Herman Amaya, Dennis Crounse [68]	12 December 2002	Mould inserts manufactured from metal injection moulding material provide high machinability rates, time and cost savings, extended tool life, and material savings. The process involves developing cutting path programmes from CAD files, machining cavity and core inserts to predefined sizes, and processing them to transform the soft material into a dense, hardenable material.
5	WO2017070748A1	Geopolymer composite and geopolymer matrix composition	Behzad Nematollahi, Jay Sanjayan [69]	4 May 2017	The dry-mix composition allows for the formation of ambient temperature-cured SHGC without the need for a liquid activator.
6	CN106082898A	3D printed geopolymer composite material, its production and applications	Lin Xi Qiang, Li Jing Fang, Zhang Tao, Huo Liang, Li Guo You, Zhang Nan, Liao Juan, Wang Bao Hua, Ji Wen Zhan [70]	31 July 2018	Slag powder composition includes blast furnace slag, steel-making slag powder, fly ash, mine tailing sand, composite exciting agent, volume stabiliser, thixotropic agent, defoamer, and mixing water. The invention’s geopolymer composite material has good caking properties, stability, form and volume stability, providing good stability and safety for building construction.

**Table 2:** Types of mould base material with examples [81,82,83,84,85,86,87].

Mould Base Material	Example of Material
Carbon steel	1018
	1050
Alloy steel	AISI 4130
	AISI M2
Stainless steel	420
	316L
	17-4 PH
Tool steel	O-1
	A-6
	S-7
	D-2
	P-20
	H13

**Table 3:** Simulation results of mould inserts by researchers [100].

	Parameter	Time to Reach Ejection Temperature (s)	Mould Core Insert Temperature (°C)	Volumetric Shrinkage (%)	Warpage (mm)
Material					
Pure copper		8.804	28.10	1.605	0.1602
Tool steel		12.400	76.82	1.759	0.1700
Beryllium copper		9.483	41.62	1.160	0.1614

**Table 4:** Different types of resin and their properties [102].

Name	EP250	NeuKadur VGSP5	EPO 752	XD4532 or XD4533	Reshape-Express 2000™
Resin Producer	MCP HEK Tooling GmbH, Lubeck, Germany	Altropol Kunststoff GmbH, Stockelsdorf, Germany	Axson Technologies (Shanghai) Co., Ltd., Shanghai, China	Ciba Specialty Chemicals Holding Inc., Basel, Switzerland	
Density (kg/m^3^)	2	2.8	1.7–1.78	1.7 ± 0.02	1.8
Tensile strength (MPa)	67	50	49	38 ± 4	62
Compressive strength (MPa)	260	180	NA	145 ± 5	251
Flexural strength (MPa)	120	NA	88	90 ± 5	82
Deflexion temperature (°C)	250	150	195	220	234
Linear expansion (×10^6^ mm/K)	30–35	30–35	50	NA	42
Hardness	112 (Rc)	90 (Shore D)	90 (Shore D15)	90 (Shore D)	91 (Shore D)

**Table 5:** Research on epoxy materials as mould inserts for RT.

	Researchers	Epoxy Resin/Hardener	Particles/Fillers Used	Weight Percentage of Filler (wt. %)	Particle Size	Mechanical Test											
						Arithmetic Mean Roughness (Ra) (µm)	Flexural Strength (MPa)	Hardness Test (R_H_)	Thermal Conductivity (W/m·K)	Fatigue Test	Tensile Strength (MPa)	Compressive Strength (MPa)	Vickers Hardness (kgF/mm^2^)	Shore D Hardness Test	Density (g/cm^3^)	Thermal Diffusivity (mm^2^/s)	Surface Roughness
1.	Tomori et al. (2004) [110]	RP4037 (resin) RP4037 (hardener)	SiC	28.5 34.7 39.9	N/A	1.03 to 1.35	58.75 to 66.49	N/A	N/A	N/A	N/A	N/A	N/A	N/A	N/A	N/A	N/A
2.	Senthilkumar et al. (2012) [111]	Araldite LY 556 (resin)	Al	40 45 50 55 60	45–150 µm	N/A	N/A	69 to 89	3.97 to 5.39	15,786 to 734	N/A	N/A	N/A	N/A	N/A	N/A	N/A
3.	Srivastava and Verma (2015) [27]	PL-411 (resin) PH-861 (hardener)	Cu Al	1 5 8 10	N/A	N/A	N/A	N/A	N/A	N/A	<85 (pure epoxy)	Cu = 65 at 10 wt. %	Cu = 22.4 at 8 wt. %	N/A	N/A	N/A	N/A
4.	Fernandes et al. (2016) [26]	RenCast 436 (resin with Al filler) Ren HY 150 (hardener)	Al	21.4	N/A	N/A	N/A	N/A	N/A	N/A	Steel AISI P20 inserts = 20.0 ± 4.5 Epoxy resin/Al inserts = 22.0 ± 5.0	N/A	N/A	Steel AISI P20 inserts = 66 ± 3.2 Epoxy resin/Al inserts = 61 ± 1.6	N/A	N/A	N/A
5.	Khushairi et al. (2017) [112]	RenCast CW 47 (resin with Al filler) Ren HY 33 (hardener)	Brass Cu	10 20 30	N/A	N/A	N/A	N/A	Brass: 10% = 1.18, 20% = 1.21, 30% = 1.37 Cu: 10% = 1.66, 20% = 1.73, 30% = 1.87	N/A	N/A	Brass: 10% = 95.61, 20% = 93.23, 30% = 92.69 Cu: 10% = 80.83, 20% = 81.51, 30% = 73.17	N/A	N/A	Brass: 10% = 1.85, 20% = 2.01, 30% = 2.22 Cu: 10% = 1.83, 20% = 1.96, 30% = 2.08	Brass: 10% = 0.644, 20% = 0.657, 30% = 0.740 Cu: 10% = 0.837, 20% = 0.923, 30% = 1.112	N/A
6.	Kuo and Lin (2019) [113]	TE-375 (Al filled epoxy resin)	N/A	N/A	N/A	N/A	N/A	N/A	N/A	N/A	N/A	N/A	N/A	N/A	N/A	N/A	Average microgroove depth of Al-filled epoxy resin was 90.5% Average microgroove width of Al-filled epoxy resin was 98.9%

**Table 6:** Research on the effects of different geopolymer compositions on mechanical properties.

No.	Researchers	Curing Days	Curing Temperature	Material Composition	Mechanical Properties	Result
1.	Girish et al. (2017) [131]	7, 14, 28	60 °C	NaOH solution from 8 M to 14 M	Compressive strength	The greatest strength attained was 62.15 MPa at 28 days. Compressive strength ratings suggest an increase in the strength of all combinations. At 28 days, the compressive strength of the cement concrete surpassed the stiff pavement’s minimum compressive strength requirement (40 MPa).
2.	Girish et al. (2018) [132]	7, 28, 56	30 °C	SiO_2_/Al_2_O_3_ ratio of 3.0–3.8 Na_2_O/Al_2_O_3_ ratio of 1	Compressive strength Flexural strength Split tensile strength Modulus of elasticity Flexural strength of beams sliced from slab	The highest strength achieved was 71.78 MPa after ambient curing at 56 days. Compressive strength values indicate an increase in the strength of all mixes. At 28 days, the compressive strength of the cement concrete exceeded the rigid pavement’s minimum compressive strength requirement (40 MPa).
3.	Izzati et al. (2020) [133]	3	FA and slag at 27 °C Kaolin at 80 °C	1.0 wt. % of either FA, kaolin, slag geopolymer particles in Sn-0.7Cu	Hardness	Slag geopolymer in SnCu solder paste impacts on the microhardness values. Slag geopolymer particles enhanced hardness by up to 7.84 Hv.
4.	Hussein and Fawzi (2021) [134]	7, 28	40°C	Cement: fine agg.: coarse agg. with 0% 5% copper fibre and fly ash and slag: fine agg. Coarse agg. with 5% copper fibre	Compressive strength Splitting tensile strength Flexural strength	The greatest improvement in compressive strength, splitting tensile strength, and flexural strength. Copper wire fibre increases splitting tensile strength and flexural strength, and when the age of the concreate increases, the MPa increases.
5.	Hussein and Fawzi (2021) [135]	2	40 °C	MR0 and MR1 cement: fine aggregate MR2, MR3, MR4—fly ash in slag at 0.75:0.25, 0.65:0.35, and 0.55:0.45	Compressive strength Splitting tensile strength Flexural strength	MR1 has the greatest preliminary compressive strength. Geopolymer mix MR4 has the highest mechanical properties. In splitting tensile strength and bending strength tests, fibre addition produces better results.

**Table 7:** Hardened properties of M10 mix [132].

Curing Period in Days	Compressive Strength (MPA)	Flexural Strength (MPa)	Split Tensile Strength (MPa)	Modulus of Elasticity (MPa)	Flexural Strength of Beams Sliced From Slab
7	45.22	3.85	-	-	4.05
28	56.41	4.63	3.96	37,471.44	4.95
56	71.78	5.42	4.96	38,197.20	5.22

**Table 8:** Research on the effects of various proportions of sodium silicate/sodium hydroxide and fly ash/alkaline activators on the mechanical properties.

No.	Researchers	The Ratio of Sodium Silicate/Sodium Hydroxide	The Ratio of Fly Ash/Alkaline Activator	Curing Temperature and Days	Mechanical Properties	Result
1.	Morsy et al. (2014) [136]	0.5, 1.0, 1.5, 2.0, 2.5	2.5	80 °C	Compressive strength Flexural strength	Curing time has a direct correlation with the increase in compressive and flexural strength.
2.	Liyana et al. (2014) [137]	1.0, 1.5, 2.0, 2.5	1.0, 1.5, 2.0, 2.5	Room temperature for 24 h	Flexural strength	Fly ash/alkaline activator yielded the highest flexural strength at ratio 2.0. Maximum flexural strength is achieved with a 2.5 sodium silicate to sodium hydroxide ratio.
3.	Bakri et al. (2011) [138]	0.5, 1.0, 1.5, 2.0, 2.5, 3.0	1.5, 2.0, 2.5	70 °C for 24 h	Compressive strength	When combined with sodium silicate and sodium hydroxide, fly ash and alkaline activator may boost concrete’s compressive strength.
4.	Nis (2019) [139]	1, 1.5, 2, 2.5	180	Ambient curing 6 ± 4 °C for 26 days Delayed oven-curing at 70 °C for 48 h	Compressive strength	As the ratio of alkali activators increased, the compressive strength of the specimens dropped at 14 M.
5.	Abdullah et al. (2021) [140]	0.5, 1.0, 1.5, 2.0, 2.5, 3.0	1.5, 2.0, 2.5	40–80 °C for 24 h	Compressive strength	The maximum compressive strength at 60 °C is achieved with a ratio of 2.0 fly ash to alkaline activator, and a ratio of 2.5 sodium silicate to sodium hydroxide.

**Table 9:** Research on the effect of the molarity of NaOH on mechanical properties.

No.	Researchers	Curing Days	Curing Temperature	NaOH Molarity	Material Composition	Mechanical Properties	Result
1.	Bakri et al. (2011) [142]	1, 2, 3, 7	70°C	6 M 8 M 10 M 12 M 14 M 16 M	Fly ash Sodium hydroxide Sodium silicate	Compressive Strength (MPa)	12 M shows the highest compressive strength reached on the seventh day. The highest compressive strength was achieved on the third day of curing.
2.	Gum et al. (2013) [143]	1, 3, 7, 14, 28, 56, 91 (7 classes)	Oven: 60 °C for 24 h Air: 20 °C for 24 h	6 M 9 M 12 M	Fly ash Sodium hydroxide Sodium silicate	Compressive Strength (MPa)	Compressive strength and early strength both seemed to improve with increased NaOH molarity, which was employed as the alkaline activator. 9 M and 12 M NaOH increased the strength by 45 MPa and 46 MPa after curing for 56 days.
3.	Lee et al. (2013) [144]	3, 7, 14, 28, 56	17 °C, 28 °C	4 M 6 M 8 M	Fly ash, slag Sodium hydroxide Sodium silicate Water glass Sand	Compressive Strength (MPa)	The molarity of NaOH was increased while alkali activator duration was decreased due to the amount of slag and water glass. The amount of slag was increased 25% and 30% at 28 days while the amount of slag decreased after 56 days of curing due to crack evolution.
4.	Rathanasalam et al. (2020) [145]	3, 7, 28	60 °C	10 M 12 M 14 M	5%, 10%, and 15% UFGGBFS replaced fly ash, with crushed stone or copper slag	Compressive Strength (MPa)	14 M NaOH concentration has the maximum compressive strength.
5.	Khan et al. (2021) [146]	28	40 °C, 50 °C, 60 °C, 70 °C	8 M 10 M 12 M 14 M	Fly ash Copper slag Crusher dust	Compressive Strength (MPa)	To achieve maximum strength, the SS/SH was maintained at 142.4, and the molarity of NaOH was maintained at 1414 M.

## Data Availability

Not applicable.
